# Can authorities appreciably enhance the prescribing of oral generic risperidone to conserve resources? Findings from across Europe and their implications

**DOI:** 10.1186/1741-7015-12-98

**Published:** 2014-06-13

**Authors:** Brian Godman, Max Petzold, Kathleen Bennett, Marion Bennie, Anna Bucsics, Alexander E Finlayson, Andrew Martin, Marie Persson, Jutta Piessnegger, Emanuel Raschi, Steven Simoens, Corinne Zara, Corrado Barbui

**Affiliations:** 1 Department of Laboratory Medicine, Division of Clinical Pharmacology, Karolinska Institutet, Karolinska University Hospital Huddinge, SE-141 86 Stockholm, Sweden; 2 Strathclyde Institute of Pharmacy and Biomedical Sciences, University of Strathclyde, Glasgow, UK; 3 National Institute for Science and Technology on Innovation on Neglected Diseases, Centre for Technological Development in Health, Oswaldo Cruz Foundation (Fiocruz), Rio de Janeiro, Brazil; 4 Centre for Applied Biostatistics, Occupational and Environmental Medicine, University of Gothenburg, Gothenburg, Sweden; 5 Department of Pharmacology & Therapeutics, Trinity Centre for Health Sciences, St James Hospital, Dublin 8, Ireland; 6 Public Health and Intelligence Strategic Business Unit, NHS National Services Scotland, Edinburgh EH12 9EB, UK; 7 University of Vienna, Vienna, Austria; 8 Hauptverband der Österreichischen Sozialversicherungsträger, Kundmanngasse 21, A-1031 Wien, Austria; 9 Department of Primary Care Sciences, Oxford University, Oxford, UK; 10 NHS Greater Manchester Commissioning Support Unit, Salford, Manchester M6 5FW, UK; 11 Unit of Medicine Support, Public Healthcare Services Committee, Stockholm County Council, PO Box 17533, SE-118 91 Stockholm, Sweden; 12 Department of Medical and Surgical Sciences, Alma Mater Studiorum - University of Bologna, I-40126 Bologna, Italy; 13 KU Leuven Department of Pharmaceutical and Pharmacological Sciences, Herestraat 49, 3000 Leuven, Belgium; 14 Barcelona Health Region, Catalan Health Service, Esteve Terrades 30, 08023 Barcelona, Spain; 15 WHO Collaborating Centre for Research and Training in Mental Health and Service Evaluation, Department of Public Health and Community Medicine, Section of Psychiatry, University of Verona, Policlinico G.B. Rossi, Piazzale L.A., Scuro 10 37134 Verona, Italy

**Keywords:** Generics, Antipsychotics, Risperidone, Demand-side measures, Drug utilisation, Cross national study

## Abstract

**Background:**

Generic atypical antipsychotic drugs offer health authorities opportunities for considerable savings. However, schizophrenia and bipolar disorders are complex diseases that require tailored treatments. Consequently, generally there have been limited demand-side measures by health authorities to encourage the preferential prescribing of generics. This is unlike the situation with hypertension, hypercholaesterolaemia or acid-related stomach disorders.

The objectives of this study were to compare the effect of the limited demand-side measures in Western European countries and regions on the subsequent prescribing of risperidone following generics; to utilise the findings to provide future guidance to health authorities; and where possible, to investigate the utilisation of generic versus originator risperidone and the prices for generic risperidone.

**Methods:**

Principally, this was a segmented regression analysis of retrospective time-series data of the effect of the various initiatives in Belgium, Ireland, Scotland and Sweden following the introduction of generic risperidone. The study included patients prescribed at least one atypical antipsychotic drug up to 20 months before and up to 20 months after generic risperidone. In addition, retrospective observational studies were carried out in Austria and Spain (Catalonia) from 2005 to 2011 as well as one English primary care organisation (Bury Primary Care Trust (PCT)).

**Results:**

There was a consistent steady reduction in risperidone as a percentage of total selected atypical antipsychotic utilisation following generics. A similar pattern was seen in Austria and Spain, with stable utilisation in one English PCT. However, there was considerable variation in the utilisation of generic risperidone, ranging from 98% of total risperidone in Scotland to only 14% in Ireland. Similarly, the price of generic risperidone varied considerably. In Scotland, generic risperidone was only 16% of pre-patent loss prices versus 72% in Ireland.

**Conclusion:**

Consistent findings of no increased prescribing of risperidone post generics with limited specific demand-side measures suggests no ‘spillover’ effect from one class to another encouraging the preferential prescribing of generic atypical antipsychotic drugs. This is exacerbated by the complexity of the disease area and differences in the side-effects between treatments. There appeared to be no clinical issues with generic risperidone, and prices inversely reflected measures to enhance their utilisation.

## Background

Health authorities across Europe are increasingly struggling to fund growing drug volumes and their associated costs within available resources, as a result of ageing populations and new premium-priced drugs [1,2]. This is already resulting in some countries no longer funding new premium-priced drugs, which is not in the best interest of any stakeholder group [1-5]. Improved knowledge of pharmacogenomics, leading to improved management of patients with improved targeting of treatments, is one way forward. However, there is a still an appreciable number of challenges to address before such approaches become routine [3].

In the meantime, there are considerable opportunities for authorities across Europe to realise appreciable savings from the increased use of low-cost generics [1]. The availability of generic risperidone provides a further opportunity for authorities to achieve considerable savings. This is because worldwide sales of atypical antipsychotic drugs were over $US 5 billion per year in the early 2000s, reaching $14.6bn in the US alone in 2009 [6,7]. In addition, medicine costs can be an appreciable component of the overall cost of treating patients with schizophrenia, as pharmacological treatments represent the backbone of managing these patients [8-11].

We acknowledge that there is continuing debate about the relative merits of atypical versus typical antipsychotics in the management of patients with schizophrenia [12-16]. Recent studies have suggested that pharmacological treatments should be tailored, in view of the considerable variation in their effectiveness between individual patients [12,17-20]. In addition, there are also considerable differences in side-effects between the different atypical antipsychotic drugs, including weight gain, hyperlipidaemia and type 2 diabetes [12,17,19]. The risk of QT prolongation and subsequent arrhythmia-related events, i.e. torsade de pointes (TdP) and sudden cardiac death, has also become increasingly important [21,22]. Previously, atypical antipsychotic drugs were generally perceived as having a more favourable safety profile in terms of cardiac and extrapyramidal side-effects. However, this is changing, with post-marketing studies and meta-analyses challenging the definition of typical (first generation) or atypical (second generation) antipsychotic drugs [18,23-27]. Recent studies have also shown that the risk of mortality in patients with schizophrenia is highest with quetiapine and lowest with clozapine [28]. However, there have been concerns regarding patient selection in this cohort study. Haloperidol and risperidone had slightly lower adjusted hazard ratios than quetiapine [28].

However, other authors believe the modest health gains achieved with atypical antipsychotic drugs reported in the literature do not adequately reflect the improvement in the quality of life perceived by patients, clinicians or carers [29]. This has resulted in the increasing use of these drugs in recent years, which is likely to continue despite safety concerns [30-37].

As a result, the introduction of generic atypical antipsychotic drugs should be welcomed by European authorities in order to save costs. However, it is recognised by health authorities that schizophrenia and bipolar disorders are complex diseases to treat compared with hypercholesterolaemia, hypertension or acid-related stomach disorders, for instance. In addition, atypical antipsychotic drugs cannot be considered as a single class, because of the heterogeneity of their pharmacological activities. This is unlike the situation for proton pump inhibitors (PPIs), renin-angiotensin inhibitor drugs or statins [1,38-45]. In view of this, as mentioned, there is a greater need to tailor treatments to individual patients. This complexity has resulted in limited demand-side initiatives by national and regional health authorities across Europe to preferentially encourage the prescribing of oral risperidone versus patented atypical antipsychotic drugs once generic risperidone became available [46-48]. Limited measures included physician prescribing quotas for low-cost medicines in Belgium, advice to psychiatrists to consider preferentially starting patients on generic atypical antipsychotic drugs where pertinent in Scotland, and prescribing restrictions for long-acting risperidone injections in Austria and Belgium [46-48].

Consequently, the principal objective of this study was to compare and contrast the effect of the limited demand-side measures instigated by Western European countries and regions to enhance the prescribing of risperidone versus patented atypical antipsychotic drugs once oral generic risperidone became available. A secondary objective was to utilise the findings to provide guidance to health authorities regarding potential measures they could consider to enhance the prescribing of generic atypical antipsychotic drugs if this is practical and feasible. This is because we would expect to see limited change in the utilisation of risperidone following generics with limited demand-side measures and the recognised need to tailor pharmacotherapy. This builds on previous findings across a range of classes, including antidepressants, PPIs, renin-angiotensin inhibitor drugs and statins [1,38-41,47,49,50]. We also investigated the utilisation of generic versus originator risperidone because a universally low utilisation of generic risperidone would represent concerns with generics among either patients or physicians, or both. Finally, we investigated prices for generic risperidone versus pre-patent loss prices to provide guidance to countries that still have high prices for generics.

Only Western European countries and regions were chosen for analysis as generic atypical antipsychotic drugs have been available for a longer time in Central and Eastern European countries [6].

## Methods

We principally undertook a segmented regression analysis of retrospective time-series analysis to assess the effect of various initiatives in Belgium, Ireland, Scotland and Sweden following the introduction of generic risperidone [51]. The xtmixed command in Stata (version 12) (StataCorp, College Station, Texas, USA) was used to fit a linear random coefficient model with country-specific intercepts. At the time of introduction of generic risperidone into each country, a random shift in intercepts and slopes was allowed to estimate the effect of the introduction. Data on the number of monthly reimbursed prescriptions within each country’s health service for all patients prescribed at least one atypical antipsychotic drug (N05AH03 to 06, N05AL05, N05AX08, N05AX011 to 13) [52] up to 20 months before and up to 20 months after the availability of generic risperidone was included. Clozapine was not included in the analysis as it is generally reserved for patients not responding to other atypical antipsychotic drugs because of its side-effect profile [46,53-55]. Ziprasidone (N05AE04) was also not included. This was in view of its different classification and limited utilisation in practice in a number of European countries, including Sweden [56].

A retrospective observational study of the same population dispensed at least one atypical antipsychotic drug was also undertaken in Austria and one of the regions in Spain (Catalonia) from January 2005 (Austria) or January 2006 (Spain) to the end of 2010 (Austria) and September 2011 (Spain). This was because generic risperidone was already available in Austria and Spain in July 2004 and by January 2006 respectively, but only became available later in the four chosen European countries: Ireland in December 2007, Scotland in April 2008, and Belgium and Sweden in January 2009. A retrospective observational study was also undertaken in one English primary care organisation, Bury Primary Care Trust (PCT), between November 2009 and October 2011. The objective was to assess the influence of a request to psychiatrists to consider oral risperidone as first line treatment in new or other suitable patients, where appropriate, now it was available as a generic.

Finally, retrospective observational studies were undertaken on the utilisation of long-acting risperidone injections versus total risperidone (N05AX08) [52], which was available throughout the study period, as well as paliperidone (N05AX13) before and after the availability of generic oral risperidone.

The European countries chosen provide a range of differences in geographical location, population size, different approaches to the financing of health care, and different approaches to the pricing of generics and to enhancing the utilisation of generics versus originators [39,40], which is in line with recommended guidance [57].

Only administrative databases were used in each country to assess the utilisation and expenditure patterns of the atypical antipsychotic drugs. This is because the perspective of the study was that of health authorities, and they typically have the greatest knowledge concerning existing and planned initiatives and reforms in their countries. The databases, which are regularly audited, are included in Box 1. Box 1 also contains details of patients included within the national health service of each country. This typically includes 100% or close to 100% of the population unless stated (Ireland), given the principles of equity and solidarity within European healthcare systems. There are also typically limited patient co-payments.

The utilisation of the different atypical antipsychotics was calculated in terms of defined daily dose (DDD), which is defined as ‘the average maintenance dose of a drug when used in its major indication in adults’, as this measure is recognised as the international standard to assess utilisation patterns within and between countries [58]. The only exception was Bury PCT, where utilisation was measured in terms of prescription items, which is the typical metric used to assess utilisation patterns in England [59]. 2011 DDDs were used in line with international guidance [58,60,61].

Separate retrospective observational studies were conducted in Belgium, Scotland and Sweden, again using an interrupted time-series methodology. The objective was to assess whether the changes in risperidone utilisation patterns after the introduction generic risperidone in these three countries were significant [46,48,56].

Subsequently, risperidone utilisation in Belgium, Ireland, Scotland and Sweden was converted into a percentage of total selected atypical antipsychotic utilisation (DDD basis) before and after the availability of generic risperidone (time 0). The objective was to enable meaningful comparisons between the four countries, factoring in differences in population sizes, time when generic risperidone became available, and differences in their database characteristics (Box 1). Utilisation patterns and calculations were verified with the relevant co-authors to enhance the robustness of the study findings.

The percentage of oral risperidone dispensed as generics was also calculated in Belgium, Ireland, Scotland and Sweden. We would expect to see considerable differences in utilisation rates between countries in view of the different policies in each country regarding encouraging the utilisation of generics versus originators [39,40,62-65]. However as mentioned, a universally low utilisation of generic risperidone would reflect general stakeholder concerns with generic risperidone.

The percentage reduction in expenditure per DDD for oral generic risperidone versus pre-patent loss originator prices was also calculated in Belgium, Ireland, Scotland and Sweden. We chose to compare relative reductions rather than actual prices for generic risperidone as the price components can vary in each country (for example, there are variations in the extent of VAT and relative wholesaler margins), and this approach also avoids currency conversions, both of which can make cross-country price comparisons difficult, especially during times of economic difficulty. In addition, prices of initial or all generics in an appreciable number of European countries are based on pre-patent loss prices [39,40,62,64,65], and the time periods for the availability of generic risperidone varied considerably between the countries and regions studied. We also did not factor inflation into the calculations because the trend in most European countries is to reduce prices when pharmaceutical expenditure exceeds target budgets [39,66] and, as mentioned, prices of generics in a number of European countries are based on pre-patent loss prices. This is in line with previous studies [39,40,64]. We would again expect to find considerable differences in the prices of generic risperidone between countries, because of the different pricing initiatives and differences in the attractiveness in the generic market [1,38,39,62,64,67].

Finally, we calculated the influence of the availability of generic risperidone on subsequent atypical antipsychotic expenditure where possible.

No ethics approval was needed or obtained because only aggregated drug utilisation data was used, without access to specific patient data.

## Results

**Figure 1:**
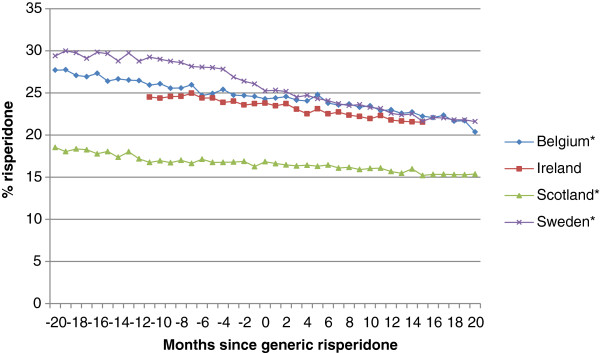
**Percentage utilisation of risperidone (defined daily dose (DDD) basis) versus selected atypical antipsychotic drugs among four European countries.** NB. *= No statistical difference in the rate of risperidone utilisation before and after the availability of generic risperidone in separate country analyses.

There was a consistent steady reduction in the utilisation of risperidone as a percentage of total selected atypical utilisation in all the four countries over time following the introduction of generic risperidone (Figure 1).

There were significant differences in the rate of utilisation of risperidone between the four countries before generic risperidone was launched (Table 1, initial slope). However, there was less variation in the utilisation of risperidone between the four European countries after generic drugs became available (Figure 1, Table 1). The average decline in the percentage of risperidone versus the other selected atypical antipsychotic drugs persisted after generic risperidone was introduced but to a lesser extent, with an initial average drop of −0.0774 and a change in slope from −0.144% to −0.00548% per month (Table 1). However, this combined change in the slope after month 0 was not statistically significant.

**Table 1:** Characteristics of the utilisation of risperidone after generic availability (month zero)

**Consolidated atypical antipsychotics following generic risperidone**		
	**Coefficient value (95% CI)**	***P*-value**
Initial intercept	22.70 (18.58 to 26.82)	<0.001
Change in intercept at month 0	−0.0774 (−1.080 to 0.925)	0.880
Initial slope	−0.144 (−0.158 to -0.130)	<0.001
Change in slope after month 0	−0.00548 (−0.0545 to 0.0436)	0.827

There was variation between the four countries in the rate of decline in the utilisation of risperidone after the introduction of generic risperidone (month 0). Sweden had the fastest decline, while Scotland, already having the lowest levels of risperidone utilisation, had the slowest decline (Table 2). However when combined, there was no statistically significant change in risperidone utilisation patterns following the introduction of generics (Table 1). There was also no significant change in the utilisation of risperidone after the introduction of generic risperidone in separate single country studies conducted in Belgium, Scotland and Sweden [46,48,56].

**Table 2:** Slope of risperidone utilisation between the four European countries after generic risperidone became available (month 0)

**Country**	**Slope**^**a**^
Belgium	−0.165
Ireland	−0.143
Scotland	−0.075
Sweden	−0.194

^a^A slope of −0.165 = a drop of 0.165%/month in the utilisation of risperidone as a percenage of all selected atypical antipsychotics following the introduction of generic risperidone.

**Figure 2:**
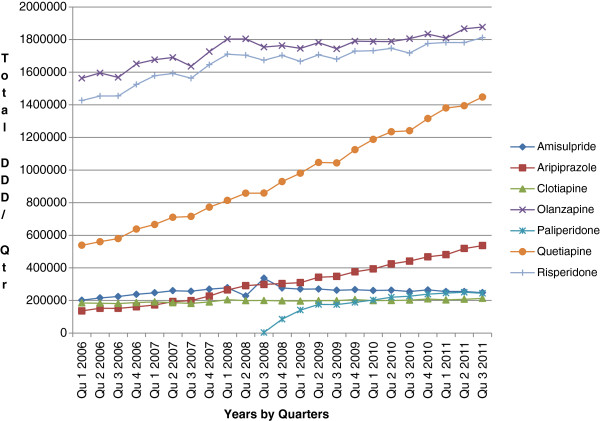
**Utilisation of selected atypical antipsychotic drugs in Catalonia, Spain, in defined daily doses (DDD) on a quarterly basis from January 2006 to end of September 2011. **NB. Generic immediate release (IR) quetiapine became available in Spain from 2008 but generic extended release (ER) quetiapine only became available in September 2011.

**Figure 3:**
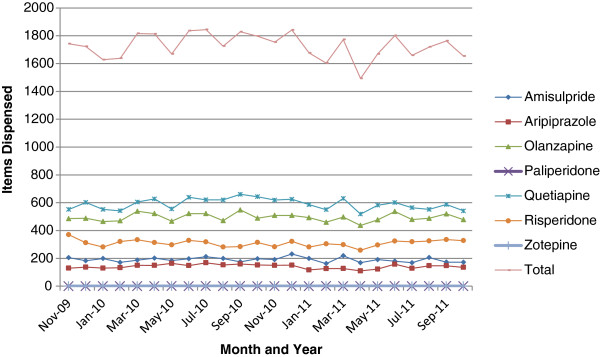
Utilisation of atypical antipsychotic drugs in Bury Primary Care Trust (PCT) November 2009 to October 2011.

A similar pattern was also seen in Austria (Table 3) and Spain (Catalonia) (Figure 2). In Spain, utilisation of risperidone declined from 35% of selected atypical antipsychotic drugs in 2006 (DDD basis) to 28% by the third quarter of 2011 (Figure 2).In Bury PCT, the prescribing of risperidone averaged between 16% and 21% of the selected atypical antipsychotics dispensed between November 2009 and October 2011 (Figure 3). However, there was no recognised pattern to the prescribing of risperidone, with its utilisation varying randomly between the months. Again, utilisation of selected atypical antipsychotic drugs was dominated by olanzapine and quetiapine.

**Table 3:** Utilisation of selected atypical antipsychotics in Austria as a percentage of total atypical antipsychotic use between 2005 and 2010 (defined daily dose basis)

**Atypical antipsychotic**	**2005**	**2006**	**2007**	**2008**	**2009**	**2010**	**% Change**
Risperidone	31.6	29.9	28.6	26.9	25.5	23.8	-25
Amisulpride	8.4	7.1	6.7	6.3	5.9	5.5	-35
Aripiprazole	2.5	5.4	7.2	9.1	10.6	11.8	376
Olanzapine	36.6	33.3	31.3	29.6	28.0	26.1	-29
Quetiapine	18.6	22.5	24.8	27.0	29.6	32.7	75
Paliperidone	0.00	0.00	0.00	0.02	0.04	0.03	NA
Zotepine	2.2	1.8	1.4	0.9	0.4	0.1	

NA, not applicable.

There was variation between the various European countries and regions in the utilisation of long-acting risperidone injections as a percentage of total risperidone. This ranged from an average of 6% to 7% in Scotland to 18.5% to 20% in Sweden, and over 20% in Belgium, before reducing in later months in Belgium following tightening of the prescribing regulations versus oral risperidone [48]. There was generally low utilisation of paliperidone in all the countries and regions studied (Table 4), despite paliperiodone being available before oral generic risperidone in the four principal countries.

**Table 4:** Maximum utilisation of paliperidone (oral and injectable) as a percentage of total selected antipsychotic utilisation in the various European countries and regions

**Country**	**Paliperidone (maximum), %**
Austria	0.04
Belgium	4.8
Bury PCT	0.07
Ireland (GMS population)	1.01
Scotland	0.03
Spain (Catalonia)	3.99
Sweden	1.5

GMS - General Medical Services.

There was also considerable variation in the utilisation of oral generic versus originator risperidone by the end of the study period in each of the four principal countries (Table 5). Similarly, there was considerable variation between the four countries in the price reduction of oral generic risperidone (expenditure/DDD) versus pre-patent loss prices by the end of the study period.

**Table 5:** Percentage utilisation of oral generic risperidone versus total risperidone (DDD basis) and percentage reduction in expenditure per defined daily dose for oral generic risperidone versus pre-patent loss prices by the end of the study period in each country

**Country**	**Utilisation of generic risperidone, %**	**Price reduction, %**
Belgium	52	59
Ireland	14	28
Scotland	98	84
Sweden	96	80

In both Scotland and Sweden, the high utilisation of generic risperidone at low prices (Table 5) resulted in expenditure for atypical antipsychotic drugs increasing at a lower rate than utilisation. Utilisation of the selected atypical antipsychotic drugs in Scotland increased by 53% between 2005 and 2010, but expenditure increased by only 42%. In Sweden, utilisation increased by 20% after the introduction of oral generic risperidone until August 2011, with expenditure increasing by only 13%.

## Discussion

As expected, there was no increase in the utilisation of risperidone compared with the other atypical antipsychotic drugs after the introduction of generic risperidone in either Belgium, Ireland, Scotland or Sweden (Figure 1), or in Bury PCT (Figure 3). In fact, if anything the reverse was seen, with increased prescribing of patented atypical antipsychotics in the four countries (Figure 1). A similar picture was also seen in Austria and Spain (Table 3; Figure 2) with generic extended release (ER) quetiapine not being available in Spain until near the end of the study. However, there were significant differences in the rate of decline in risperidone utilisation between the four countries before generic risperidone was launched (Table 1). However, there was less variation in the rate of decline after generic risperidone became available (Figure 1; Table 1), with the combined decline in risperidone utilisation falling to −0.00548% per month from −0.144% per month (Table 1). The rate of decline was greater in Sweden than Scotland (Table 2). However, there was overall a reasonable consistency between the four countries, irrespective of their characteristics [39,68], reflected by the lack of a statistically significant change in slope after month 0 (Table 1). This was also no statistically significant difference in the rate of risperidone utilisation after generic risperidone became available in the separate analyses conducted in Belgium, Scotland and Sweden [46,48,56].

The consistent findings between the seven European countries and regions, including Austria (Table 3), Spain (Figure 2) and Bury PCT (Figure 3), regarding risperidone utilisation following the introduction of generics would suggest that following generic availability, there was no increased prescribing of oral risperidone for new patients, for whom risperidone could be one of the treatment options. However, we cannot say this with certainty without analysing patient-specific data. No increased prescribing of risperidone following introduction of generics (Figures 1, 2 and 3; Tables 1 and 3) may reflect the advice from organisations such as the National Institute for Health and Care Excellence in the UK and from various published studies that treatment of patients with schizophrenia should be individualised to maximise patient outcomes [17-19,69,70]. The growing utilisation of the other atypical antipsychotics, especially quetiapine and aripiprazole, in the various countries following the introduction of generic risperidone (Figures 1 and 2; Table 3) may reflect the marketing activities of the manufacturers of patented atypical antipsychotic drugs including ER quetiapine in Spain, influencing the choice of antipsychotic drug prescribed [46,48,71-73]. However, it is more likely to reflect the recognised weight neutrality with aripiprazole versus olanzapine and risperidone, as well as the effectiveness of aripiprazole and quetiapine ER in treating patients with major depressive disorders who have had an incomplete response to antidepressants, and of quetiapine ER in treating patients with bipolar depression [74-76], given the limited utilisation of patented paliperidone in recent years (Table 4). However, this remains to be elucidated in further research. There was also no substantial increase in the utilisation of long-acting risperidone in the four principal countries following the introduction of oral generic risperidone. If anything, the reverse was seen in Belgium in recent years, as reimbursement is denied if the medical adviser appointed by the patient’s insurer is not satisfied with the rationale provided by the physician [48].

The findings also potentially suggest there is no ‘spillover’ or cross-transfer of learning in practice from one disease area to another to produce changes in physician prescribing habits, that is, no crossover of learning to increase the prescribing of generics when available as seen with the PPIs, renin-angiotensin inhibitor drugs and statins [1,38-41,43-45,59,77]. We believe this is an important finding from this research. However, this finding is tempered by the recognised need to tailor pharmacological treatment for patients with schizophrenia or bipolar disease, especially with regard to issues such as weight gain and effectiveness in different patient populations, as well as reluctance among physicians to switch treatments when patients are stable on a particular atypical antipsychotic drug.

We believe a second important finding is that in some disease areas it is difficult for health authorities to encourage the preferential prescribing of multiple sourced versus patented drugs, apart from introducing measures such as prescribing restrictions for different formulations of a molecule [48]. This illustrated by limited initiatives in any of the seven countries and regions to enhance the prescribing of oral risperidone following the introduction of generics. This is unlike the situation for the PPIs, renin-angiotensin inhibitor drugs and the statins [1,38,40,42,77]. We believe, based on our findings (especially those from Bury PCT following its activities (Figure 3) when recently it was very successful in significantly enhancing the prescribing of generic losartan versus patented ARBs (angiotensin receptor blocker) for treating hypertension with multiple demand-side measures [59]), that the influence of measures such as prescribing guidance or guidelines highlighting the preferential prescribing of generic atypical antipsychotic drugs as first line treatments may be limited. This is especially the case if there is a good clinical rationale for prescribing a patented product including concerns with weight gain. Additional measures could include instigating reimbursement restrictions for oral patented atypical antipsychotics, which is similar to the situation for long-acting risperidone injections in Austria and Belgium [47,48]. However, such measures may again be difficult to implement, given the subjective nature of choosing pharmacological treatment options to maximise patient outcomes in these complex disease areas, and may even be counterproductive.

The considerable variation between European countries in the prescribing of oral generic risperidone versus originators (Table 5) reflects the different policies in these countries to encourage use of generics. The high rates seen in Scotland and Sweden suggest that there are no problems with generic risperidone in clinical practice. This is no doubt enhanced by the strict regulations for granting marketing authorisation for generics in Europe, with authorities removing generic products where concerns exist [63,64,78]. Consequently, the differences are down to different demand-side measures between the four countries. The high utilisation of oral generic risperidone in Scotland reflects generally high voluntary INN (International Non-proprietary Name) prescribing rates across classes. This starts with extensive physician education in medical school to prescribe by INN, which is followed up in ambulatory care through pharmacists working for the Health Boards monitoring the prescribing of drugs [40,46,49]. The high rates in Sweden reflect the instigation of compulsory generic substitution, including risperidone, apart from in a limited number of cases [39,40,56,77,79,80]. We believe the high voluntary INN prescribing rates in the UK provides guidance to other countries. This is because such activities reduce patient confusion once multiple sources become available, especially if patients are dispensed different branded generics with different names on each occasion, without adequate explanation. This can happen in Sweden with compulsory generic substitution, apart from a limited number of situations authorised by the Medicine Product Agency [81], and more recently with monthly auctions as the cheapest branded generic secures an appreciable proportion of prescriptions for the molecule the following month [1,38]. The dispensing of different branded generics on each occasion can possibly cause confusion and concern if patients do not receive adequate information about their medicines [82]. This can potentially result in either duplication of medicines, or alternatively, in patients not taking their prescribed treatments as directed, which could be problematic [82,83]. INN prescribing, apart from a limited number of well-known situations, is one way to address this [49,80,84,85].

There were also appreciable differences between countries concerning the price of generic risperidone (Table 4). This reflects the different policies between the four countries with regard to enhancing the utilisation of generics, as well as their different pricing policies. The considerable price reduction for generic risperidone in Scotland, which is similar to those for other generics, follows recent reforms in the UK to enhance transparency in the cost of producing generics, as well as discounts offered by manufacturers to wholesalers and pharmacists to preferentially dispense their generic [41,49]. The price reduction in Sweden, which is also similar to those for other generics, is a result of the introduction of compulsory generic substitution with the lowest priced molecule [1,38,80]. Generic prices are likely to fall further in Sweden with the recent introduction of monthly auctions, with the manufacturer who wins the auction being guaranteed a considerable proportion of dispensed generics the following month [1,38]. The more modest price reduction for generic risperidone in Belgium reflects the current situation, where generic companies only have to lower their prices to the reference price level to be reimbursed. This was only 16% versus pre-patent loss prices until 2002, 20% until 2003, 26% until 2005, and is currently 31% [48,68,85]. The high prices for generics in Ireland reflect the limited measures to date to reduce these, although this is now changing [39,86]. These findings are consistent with other research showing that the lowest prices for generics in Europe are seen in countries with the greatest market share [62,63,67]. Consequently, measures to increase the attractiveness of the generic market, as well as enhance the transparency in their pricing, as seen in Sweden and the UK, provide guidance to countries seeking ways to achieve further savings from the use of generics. This is especially the case where it is difficult to encourage the preferential prescribing of generics versus patented products, for example, atypical antipsychotic drugs.

We are aware there are a number of limitations with this study. This includes no access to patient data to assess whether there has been an increase in the prescribing of risperidone as first line treatment since the introduction of generics. In addition, there is no knowledge of the prescribed indications, especially with risperidone being the only atypical antipsychotic drug currently licensed for asymptomatic treatment in patients with dementia. However, the consistent continued decline in the utilisation of risperidone following the introduction of generics, coupled with increased utilisation of patented atypical antipsychotic drugs (Figures 1 and 2; Table 3), suggests there has been no increase in the prescribing of risperidone following generics. This may be enhanced by increased awareness of the lack of effect on weight with aripiprazole, and the effectiveness of aripiprazole and quetiapine ER in major depressive disorders. We have also not assessed whether there are any differences in outcomes between oral generic and originator risperidone. Previous research findings and the continued high utilisation of generic risperidone in Scotland and Sweden (Table 5) suggest there are no problems with generic atypical antipsychotic drugs in clinical practice [6,46,49,50,87]. However, again, we cannot say this with certainty without specific patient research. Finally, we are unable to determine or comment on the extent of any polypharmacy with atypical antipsychotic drugs.

## Conclusions

Generics provide a considerable opportunity for authorities to fund increased drug volumes and new premium-priced drugs within available resources. However, there are disorders such as schizophrenia for which it is difficult to encourage the preferential prescribing of multiple sourced drugs as first line treatments. This is due to the recognised need to tailor pharmacological treatments to the individual patient in order to maximise outcomes. This belief has resulted in limited demand-side measures by the seven European countries and regions to encourage the preferential prescribing of generic versus patented atypical antipsychotic drugs, compared with the multiple measures generally instigated for the PPIs and statins following introduction of generics. We have also shown that authorities across Europe cannot rely on the transfer of learning concerning the prescribing of generics as first line treatment from one class to another in order to affect changes in the prescribing habits of atypical antipsychotic drugs. This is no doubt enhanced in this case by the need to tailor treatments and the heterogeneity of the products in the class. However, we believe that any demand-side initiatives, apart from encouraging one dosage form over another, would have only a limited effect, owing to the complexity of treating patients with schizophrenia or bipolar disease, and the recognised differences in side-effect profiles between the various pharmacological approaches. Consequently, we do not believe the authorities in any of the seven countries or regions studied are planning specific measures in the future. This decision is no doubt helped by more oral atypical antipsychotic drugs now being available as multiple sourced products, helping to lower overall drug acquisition costs.

Finally, we believe countries can learn from each other regarding potential additional ways to further enhance the prescribing of generic versus originator atypical antipsychotic drugs, and to obtain lower prices where pertinent. This includes measures such as increasing INN prescribing and greater transparency in the pricing of generics.

## Competing interests

A number of the co-authors are employed by health authorities: MB (Scotland), AM (Greater Manchester Commissioning Support Unit), MP (Stockholm County Council, Sweden), JP (HVB Austria) and CZ (Barcelona Health Region, Catalan Health Authority, Spain). AB is an advisor to MEDEV (an informal group of the pharmaceutical experts of European Health Insurance Organizations). SS holds the European Generic Medicines Association (EGA) Chair of ‘European Policy Towards Generic Medicines’. Otherwise, the authors have no other relevant affiliations or financial involvement with any organisation or entity with a financial interest in or financial conflict with the subject matter or materials discussed in the manuscript, apart from those already mentioned.

## Authors' contributions

BG, AEF, ER and CB devised the concept for the paper and produced the first and subsequent drafts. BG is the guarantor of the paper supported in the scientific content by ER and CB. MP performed the statistical analyses, and critiqued successive drafts. KB, MB, AB, AM, MP, JP, SS and CZ provided the utilisation and expenditure data for their respective countries and regions (Austria, Belgium, Bury PCT, Ireland, Scotland, Spain (Catalonia) and Sweden) as well as details of the various demand-side measures. They also critiqued successive drafts. All authors read and approved the submitted manuscript.

## Box 1 – Administrative databases used in the study [39-41,46-48,56,59,68,86,88]

• Austria: Internal data warehouse of the HVB (Hauptverband der Österreichischen Sozialversicherungsträger) – BIG – coupled with Cube HMSTAT, based on the ‘Maschinelle Heilmittelabrechnung’. This provides reimbursement data on medicines dispensed in ambulatory care in approximately 98% of the Austrian population.

• Belgium: Pharmanet, a database of reimbursed medicines dispensed in ambulatory care in Belgium. This database is maintained by the National Institute for Health and Disability Insurance and covers the whole Belgian population.

• England (Bury PCT): National Health Service Business Services Division prescription pricing database (ePACT).

• Ireland: the National Shared Services Primary Care Reimbursement Service of the Health Service Executive in Ireland (HSE-PCRS) pharmacy claims database. This database provides details on monthly dispensed medications for each individual within the GMS population. The GMS population covers approximately 30% of the population of Ireland with higher morbidity than the general population, which is reflected in their consumption of approximately 65% of total pharmaceutical expenditure in Ireland.

• Scotland: NHS National Services Scotland Corporate Warehouse, covering the entire population in Scotland.

• Spain (Catalonia): DMART (Catalan Health Service) database, covering the public system in Catalonia.

• Sweden: National Swedish Pharmacy Register covering the entire Swedish population.

## Pre-publication history

The pre-publication history for this paper can be accessed here:

http://www.biomedcentral.com/1741-7015/12/98/prepub
